# A Semi-Mechanistic Prediction of Residence Time Metrics in Twin Screw Granulation

**DOI:** 10.3390/pharmaceutics13030393

**Published:** 2021-03-16

**Authors:** Shashank Venkat Muddu, Lalith Kotamarthy, Rohit Ramachandran

**Affiliations:** Department of Chemical and Biochemical Engineering, Rutgers—The State University of New Jersey, Piscataway, NJ 08854, USA; shashank1992.venkat@gmail.com (S.V.M.); lalith.kotamarthy@rutgers.edu (L.K.)

**Keywords:** continuous wet granulation, prediction, residence time

## Abstract

This work is concerned with the semi-mechanistic prediction of residence time metrics using historical data from mono-component twin screw wet granulation processes. From the data, several key parameters such as powder throughput rate, shafts rotation speed, liquid binder feed ratio, number of kneading elements in the shafts and the stagger angle between the kneading elements were identified and physical factors were developed to translate those varying parameters into expressions affecting the key intermediate phenomena in the equipment, holdup, flow and mixing. The developed relations were then tested across datasets to evaluate the performance of the model, applying a k-fold optimization technique. The semi-mechanistic predictions were evaluated both qualitatively through the main effects plots and quantitatively through the parity plots and correlations between the tuning constants across datasets. The root mean square error (RMSE) was used as a metric to compare the degree of goodness of fit for different datasets using the developed semi-mechanistic relations. In summary this paper presents a new approach at estimating both the residence time metrics in twin screw wet granulation, mean residence time (MRT) and variance through semi-mechanistic relations, the validity of which have been tested for different datasets.

## 1. Introduction

### 1.1. Twin Screw Wet Granulation

Wet granulation is a size enlargement process wherein a liquid solvent is added to a primary powder bed that consists of one or more components. The solvent is usually water or a polymer solution [1,2,3,4,5]. The size enlargement process occurs in the presence of binder that is added either in a dry state and mixed along with the formulation/primary powders in the granulator or dissolved in the liquid binder solvent [6,7]. The addition of the binder in the wet granulation process facilitates the formation of liquid capillary bridges that hold the primary particles together via granulation mechanisms such as nucleation, aggregation, consolidation, breakage and layering [8].

**Figure 1:**
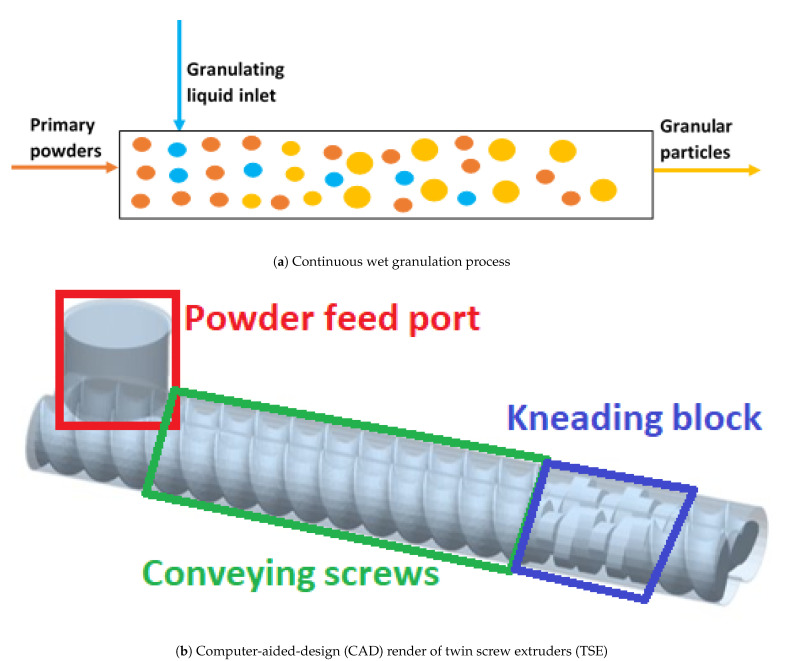
(**a**): Schematic of a typical continuous wet granulation process and; (**b**): a TSE used for continuous wet granulation process.

In recent years, there has been an accelerating shift towards continuous manufacturing, partly driven by the growing need for industries to reduce the time-to-market for products, while maintaining strict quality requirements. This has resulted in extensive investigation of continuous wet granulation processes, and its associated processing equipment. Wet granulation is carried out continuously in twin screw extruders (TSE) by employing powder feeders in order to maintain steady inlet flow into the system, and the liquid (with or without a polymer binder dissolved in it) is pumped at the desired flow rates into the mixers in a controlled flow ratio with respect to the primary powders. The liquid is discharged through nozzles into the granulator in order to achieve uniform and proper wetting of the powder particles. A typical continuous wet granulation setup and a computer-aided-design (CAD) render of a TSE, are illustrated in Figure 1a,b.

An improved process understanding and characterization of the continuous granulation unit operation necessitates mechanistic analysis of the physical processes occurring within the unit operation. The typical critical quality attributes of a wet granulation process are the exit particle size distributions, densities and the distribution of the formulation material components within the granules of different sizes. Population balance models have been extensively developed and studied in order to completely and accurately characterize number and mass distributions of various important particle traits such as size, liquid content and/or porosity [9,10]. However, they usually do not account for the initial and final segregation between the components [11]. This leads us to develop residence time distribution (RTD) models for studying mixing behavior in wet granulation. RTD studies are also useful for process design, material traceability, control and optimization.

Experimental studies are typically performed to develop and validate an RTD model, which can be further used for process model development and optimization. The RTD of continuous wet granulation systems is measured via the use of a tracer material, which tracks the movement of bulk material within the system. The tracer is added at the entrance of the granulator and then blended and distributed inside according to the conveying and mixing profile of the system. The granules exiting the system are analyzed using different techniques to quantify the RTD. The tracer content in the exiting granules can either be measured from offline samples collected or in-line measurement techniques. The concentrations of the tracer in these granules are measured using ultraviolet-visible (UV-Vis) [12] or near infrared (NIR) spectrophotometry. The RTD profiles are dependent on the effective material holdup volume inside the equipment, flow rates of the solid powder streams, the liquid-to-solid percentages (LS %ages), the rotation speed of the shaft, the configuration of the shaft elements (conveying/ kneading/ distributive elements) and the material properties such as density, compressibility and particle size of the components of the solid powder blend. The obtained time-dependent RTD profiles can then be fitted to mathematical relations either by observing the profile curve characteristics or through first-principled approaches by looking at the mechanics of the system under study.

The development of an accurate and predictive model of the RTD of a system would provide a thorough understanding of the process dynamics, and the model can be used to optimize the design space of the process. Shirazian et al. [13] presented a study where the equipment was modeled by dividing the equipment into theoretical mixing tanks and plug flow regions. The work described by Ismail et al. [14] leveraged the availability of advanced statistical tools such as artificial neural network (ANN) towards actually predicting the mean residence time (MRT) of the granulation system itself from the experimental points and additional points generated using Kriging sampling tools. The MRT itself was calculated from the RTD pulse response exit function developed and then, modeled from a Zuastz function developed by Poulesquen et al. [15,16]. However, the presented statistical model did not provide any semi-mechanistic correlations between the operational parameters and the theoretical parameters of the model.

RTD models have been extensively developed for continuous reactors in traditional chemical engineering such as continuous stirred tank reactors (CSTRs) and plug flow reactors (PFRs) dealing with mostly incompressible liquid systems and fully-developed flow profiles. These models were fitted to non- reacting systems using the experimental data of different industries such as food, pharmaceutical manufacturing and even in environmental engineering [17,18,19]. One such study by Kumar et al. has described an RTD model for a twin screw granulation (TSG) system and attempted to fit the experimental data to the model with some specified parameters [20,21]. Apart from predicting the full RTD profiles for the experimental conditions, the authors also attempted to give a physics based explanation for the tuning factors of their model. However, a model developed for a specific system needs to be re-validated (and often its parameters need to be re-calculated) for different equipment and/or different formulation.

In addition to studying the MRT and RTD profiles, researchers have also attempted to understand axial mixing in TSG. Axial mixing in continuous equipment is characterized by the variance of the RTD profiles. The mixing in radial direction is neglected due to the narrow geometries of extruders characterized by their length-to-diameter (L/D) ratios [22]. The degree of axial mixing in TSG can be estimated by determining the dimensionless Peclet number, which is the ratio between rate of transport by convection and rate of transport by diffusion/dispersion. The relationship between Peclet number and variance was originally developed by Levenspiel et al. and later Fogler showed how different boundary conditions can give rise to different correlations [23,24]. Lee et al. [25] studied RTD using a positron emission particle tracking (PEPT) technique and in their study they found that the extent of axial mixing was not effected by screw speed, powder feed rate or screw configuration. One of the drawbacks is that this study does not employ the effect of number of kneading elements. Kumar et al. [20] also studied the extent of axial mixing by measuring the variance of the RTD curves. In their study they found that screw speed, material feed rate, number of kneading discs and stagger angle had significant effects on axial mixing. They also found that interaction between screw speed and powder feed rate, and between stagger angle and number of kneading elements had a significant effect on axial mixing.

In general, processes involving wet powder flow behavior require close examination. Therefore, it is the overall aim of the researchers working in this field to develop mechanistic models accounting for the chosen vessel’s geometry (length of screws, pitch of screws, screw element configuration), its operating parameters (screw speeds and rotation direction of screws), process parameters (material feed rates and the rate of liquid addition) and the material properties (density, particle size etc.) of the constituent materials.

### 1.2. Objectives

This work proposes to further understand the physical processes governing the RTD in a TSG by developing a method to predict the central moments of RTDs, namely the MRT and variance using semi-mechanistic relations that can be extended to systems of different scales, screw configurations and process parameter ranges. The predicted values of the central moments of RTD have been compared qualitatively with the main averaged effects of individual parameters at different settings for each subset considered. In addition, the fidelity of the predictions were further validated by comparing them with the corresponding experimental points and evaluating statistical measures of goodness of fits. The historically produced twin screw wet granulation experimental data that were made available in literature by various researchers in the field were leveraged to train the semi-mechanistic models and evaluate the validity of the relations developed. It is to be noted that, in this work, the focus has been kept on having as mechanistic a model as possible for predicting the metrics across datasets and hence, the relations and tuning parameters were chosen to reflect the physics of the system.

The rest of the paper is organized as described in this paragraph. Section 2 introduces three experimental datasets used in the study that have been sourced from existing published data in literature. Section 3 concerns with the theoretical background of the paper and introduces new semi-mechanistic relations for estimating MRT and variance subject to their physical constraints. Section 4 shows the results of the study and discusses the model prediction performance for different datasets. Section 5 is a short conclusion that summarizes the paper and provides suggestions for future researchers to improve and build upon the modeling scheme presented in this work.

## 2. Materials and Methods

The RTD experimental data were collected from the published available literature [14,21,26]. The dataset collated has been described below in Table 1. The dataset has been classified based on source, equipment dimensions, varied process and equipment variables: powder feed rate (**FR**), processing screw speed (**RPM**), liquid-to-solid percentage (**LS**), screw configuration described by number of kneading elements (**NK**) and stagger angle (**SA**) between them. The table also lists the available number of points sourced from each study.

In addition to the above mentioned categories, the database also contained details on the equipment parameters such extruder shaft outer diameter, and effective length scales of operation. Lastly, the dataset also considered material properties affecting the flow in a wet granulation system, namely the bulk and true densities of the powder processing material and the density of the binder liquid fed into the system. The collation and categorization of along these parameters aided us in comparing published residence time metrics data from various experimental sources using different materials on different equipment. This mode of organization is the first step in building a more generalizable prediction model as opposed to being confined within the bounds of a chosen DOE, equipment and formulation material.

## 3. Theory and Metrics

### 3.1. Theory behind Equation Development

For this study, three new physically relevant reduced order expressions have been introduced namely, Flow factor, Holdup factor and Mixing factor. Furthermore, these reduced order expressions have been employed to formulate equations for the prediction of MRT and variance.

Flow factor: Flow factor defines the convective/ bulk flow of the powders in the axial direction. It has been found that as the feed rate is increased the residence time of powders in a TSG decreases, which has been attributed to the axial velocity component provided by the feed rate [27,28,29]. It is also known that impeller speed effects the convective capacity of the powders in TSG: as the impeller speed increases the axial velocity of powders increase thereby decreasing the MRT [25,30,31].

Holdup factor: In previous studies either dimensionless or physically significant reduced order parameters were developed in order to describe the barrel fill level in TSG systems [27]. In this study, in addition to the effects of the powder and liquid material feed rates [32], the mechanistic effects of the screw elements that is, conveying sections and kneading blocks have been incorporated too in building the relations for Holdup factor as it has been known from prior study that fraction of the section volumes are responsible for holding the particulate material [33].

**Figure 2:**
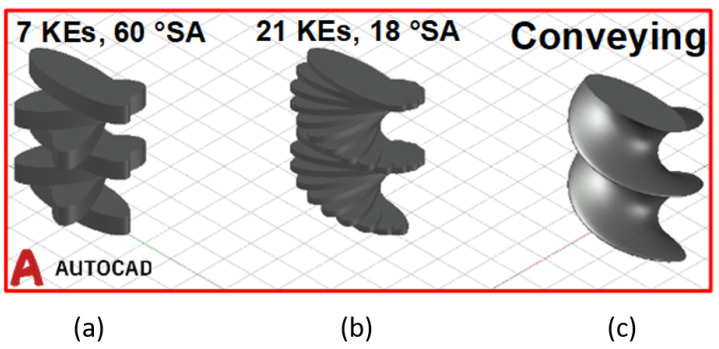
Schematic showing (**a**) kneading block of 7 elements each with thickness 3 units and **SA** 60${}^{\circ }$; (**b**) kneading block of 21 elements each with thickness 1 unit and **SA** 18${}^{\circ }$; and (**c**) conveying section of length 21 units and full turn angle 360${}^{\circ }$.

Available volume in screw sections: While it is known that the sector volume containing the stagger angle is responsible for the fill in kneading elements, the relations for conveying section are unknown as they are continual helical screws. Therefore, in this study the conveying section of a particular length and angular turn has been approximated as a kneading section of the same length where the number of kneading elements is large, the thickness of each element in the section and the stagger angle between the kneading elements are infinitesimally small. The finite element approximation schematic has been shown below in Figure 2.

Mixing factor: Mixing in flow systems can be divided into global mixing and local mixing [34]. The variance of the RTD is more appropriately described by local mixing. It was found in many mixing studies that the local mixing was enhanced with increasing shaft rotation speeds due to velocity gradients created in the transverse direction [35,36,37,38]. A previous study has shown that there is an increase in the degree of local mixing when particles entering at different times interact with each other [39]. Apart from the shaft rotation speeds, the local mixing in TSG is directly proportional to the stagger angle between the kneading elements and, inversely proportional to the number of kneading elements.

Mean residence time (MRT): Mean residence time (MRT) is defined as the average amount of time powders or a particle spends in the system of interest. In this study, this metric has been obtained from the more traditional definition in chemical engineering by dividing the material holdup by the flow rate through the system.

Variance: Most of the studies involving RTD and describing variance in a TSG have either directly correlated variance to the mixing efficiency or have described mixing via the change in Peclet Number assuming the closed-closed boundary condition approximation for TSG [24]. However, the handling of solid particulate material with size distributions in continuous operations such as feeders, blender, twin screw granulators and so forth, leads to segregation of particles continually throughout the course of flow. This causes the dispersion effects to linger outside the boundary of the system as well [40,41]; thus interfering in the measurement of the concentration of particles [42,43]. Hence in this study, the Peclet number has been modeled assuming the open-open boundary condition.

### 3.2. Formulation of the Equations for the Metrics

The expression for the net volumetric material feed-rate $FR_{vol,net}$ has been given in Equation (1):

(1) $$FR_{vol,net}=\frac{FR_{mass,powder}}{\rho_{powder}}+\frac{FR_{mass,liquid}\times LS_{ratio}}{\rho_{liquid}},$$

where $FR_{mass,powder}$ is the primary powder mass flow rate fed into the granulator, $\rho_{powder}$ is the density of the powder fed, $LS_{ratio}$ is the ratio of the mass feed rate of the liquid to the mass feed rate of the powder, and $\rho_{liquid}$ is the density of the granulation liquid. The available volume in the kneading blocks $Availvol_{knead}$, has calculated as shown in Equation (2):

(2) $$Availvol_{knead}=4\times \frac{1}{2}\times R^{2}\times NK_{knead}\times t_{knead}\times SA_{knead,deg}\times \frac{\pi }{180},$$

where *R* is the outer radius of the kneading element, $NK_{knead}$ is the number of kneading elements in the kneading block for the screw configuration, $t_{knead}$ is the thickness of each kneading element and $SA_{knead,deg}$ is the stagger angle between the kneading elements for the configuration. Based on the finite element approximation of the conveying screw as described in Section 3.1, the expression for the available volume for the conveying section $Availvol_{convey}$ has been given in Equation (3):

(3) $$\begin{array}{cc} Availvol_{convey}=[4 & \times \frac{1}{2}\times R^{2}\times NK_{convey,FE}\\ \; & \times t_{convey,FE}\times SA_{convey,FE,deg}\times \frac{\pi }{180}], \end{array}$$

where $NK_{convey,FE}$ is a big number of kneading discs making up the conveying screw, $t_{convey,FE}$ is the small thickness of each kneading disc, and $SA_{convey,FE,deg}$ is the extremely small stagger angle between the kneading elements for the finite element approximation of conveying section. From (2) and (3), the expression for the total available volume $Availvol_{total}$ has been obtained in Equation (4):

(4) $$Availvol_{total}=Availvol_{knead}+Availvol_{conv}.$$

The expression for the volumetric material displacement rate by a conveying screw of 1 lead length in the equipment $Dispvolrate_{conv,1lead}$ is given in Equation (5):

(5) $$Dispvolrate_{last}=\frac{RPM}{60}\times Availvol_{last},$$

where RPM is the shaft rotation speed in rotations-per-minute and $Availvol_{last}$ is the volume held by the last element of the shaft in the configuration.

By using the expressions developed in Equations (1) and (4), the surrogate expression for the volumetric Holdup factor Holdup has been given by the following Equation (6):

(6) $$Holdup=FR_{vol,net}^{b_{2}}\times Availvol_{total},$$

where $b_{2}$ is a tuning constant. Similarly from Equations (1) and (5), the surrogate expression for the volumetric Flow factor Flow has been computed from following Equation (7):

(7) $$Flow=FR_{vol,net}^{b_{3}}\times Dispvolrate_{conv,1lead}^{b_{4}},$$

where $b_{3}$ and $b_{4}$ are tuning constants. Lastly, the surrogate expression for the Mixing factor Mixing has been formulated as follows in Equation (8):

(8) $$Mixing=\frac{SA_{knead,deg}\times Dispvolrate_{conv,1lead}^{b_{6}}\times KB\times \pi }{NK_{perblock,knead}\times 180}.$$

Dividing Equations (6) and (7), and by multiplying by a tuning constant $b_{1}$, the semi-mechanistic expression for the mean residence time MRT has been derived in Equation (9):

(9) $$MRT=b_{1}\times \frac{FR_{vol,net}^{b_{2}}\times Availvol_{total}}{FR_{vol,net}^{b_{3}}\times Dispvolrate_{conv,1lead}^{b_{4}}},$$

with the tuning constants $b_{2}$, $b_{3}$ and $b_{4}$ subject to the following constraint (to ensure value of MRT calculated in (9) is in unit seconds in Equation (10):

(10) $$-b_{2}+b_{3}+b_{4}=1.$$

Similarly, dividing Equations (7) and (8) and multiplying by tuning constant $b_{5}$, the semi-mechanistic expression for Peclet number Pe has been derived in Equation (11):

(11) $$Pe=b_{5}\times \frac{FR_{vol,net}^{b_{3}}\times Dispvolrate_{conv,1lead}^{b_{4}}}{\frac{SA_{knead,deg}\times Dispvolrate_{conv,1lead}^{b_{6}}\times KB\times \pi }{NK_{knead}\times 180}},$$

with the tuning constants $b_{3}$, $b_{4}$ and $b_{6}$ subject to the following constraint (to ensure value of Peclet number calculated in (11) is non-dimensional) in Equation (12):

(12) $$b_{3}+b_{4}=b_{6}.$$

From the open-open boundary condition of the TSG, the normalized variance $Var_{norm}$ has been given by the following expression in Equation (13):

(13) $$Var_{norm}=\frac{2Pe+8}{{\left(Pe+2\right)}^{2}}.$$

### 3.3. Algorithm Development

**Figure 3:**
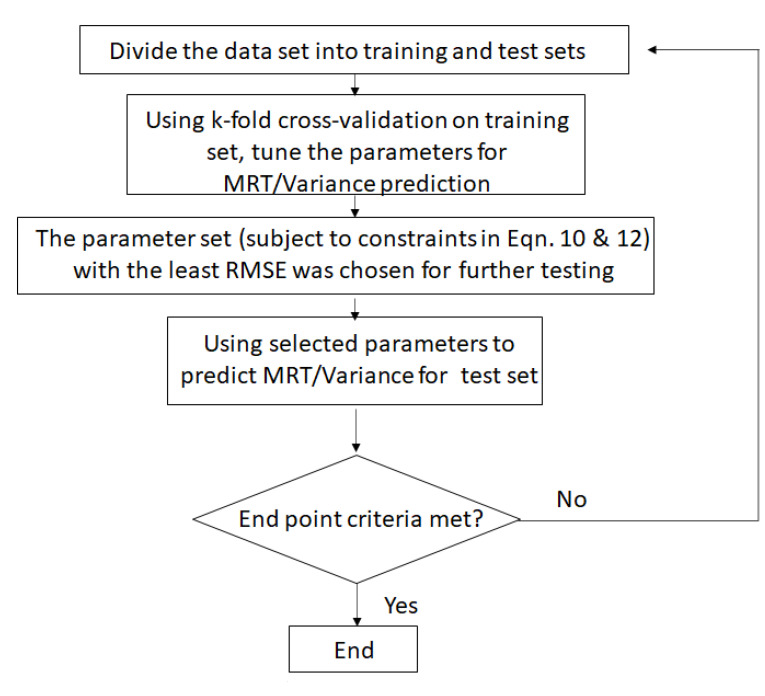
Schematic for algorithm adopted to train and validate mean residence time (MRT) and variance models on datasets.

Figure 3 shows the schematic of the algorithm developed for predicting MRT and variance. The general idea for the algorithm goes through the following steps. Initially, the experimental dataset goes through a splitting step. After the split, the larger subset (i.e., training set) is used to train the model for MRT and Peclet number (and in turn the variance) while ensuring the constraints described in Equations (10) and (12) in Section 3.2. Training is based on the developed Equations (9) and (11) as described in Section 3.2. For parameter optimization the ’fmincon’ function in MATLAB was used, the parameters resulting in predictions having the least root mean square error (RMSE) were chosen to further predict the MRT and variance for the initially divided smaller subset (test set). If the predictions on the smaller subset meet certain end point criteria, then the model is stopped. If not, the process is started again.

In order to ensure a good prediction of the model, validation of the model needs to be conducted. However, especially when we have smaller datasets, a simple validation (validation runs on a small percentage of the full dataset) might not be sufficient. Hence, in such cases cross-validation techniques need to be employed for higher degree of confidence in model predictions. For this reason, we have employed a k-fold cross-validation model. To further increase the confidence in our predictions, we initially split the experimental dataset into 2 subsets—one consisting of approximately 70–80% of the data points (Training set) and the second one comprising of the remaining points (Test set), respectively. Prior to splitting, the corresponding dataset rows were jumbled and the test set runs were randomly selected using the ’rng’ function in MATLAB with a specified seed number number to ensure that the procedure is reproducible [44]. The larger subset is used for training and the smaller one is kept aside for testing the model (unseen to the model). On the Training set the k-fold cross-validation is employed to make sure the predictions of the model were accurate.

K-fold cross-validation: In a k-fold cross-validation the parent dataset is arbitrarily divided into *k* datasets and every time the model is run k−1 datasets are used to train and 1 dataset is used to validate the model. This step is repeated *k* times such that model is validated on each of the *k* datasets. k-fold cross-validation was used to further enhance the parameter optimization process by introducing the parameter output of one iteration as an input to the next iteration.

Three different metrics were used to determine the end point criteria of the model.

i Comparison between main effects plot of the experiments and that of the model
ii Satisfactory parity plots, realistic narrow upper and lower bounds were set, and the model’s performance was determined based on how many predicted points fell between these limits; in addition the root mean square of errors RMSE too was evaluated as a statistical measure for goodness of fit
(iii) Wherever possible, the values of the tuning parameters in the model when trained on different datasets were compared, and physical interpretation was made for their ranges

The expression for the root mean square errors is given as follows in Equation (14)

(14) $$RMSE=\sum_{i=1}^{n}{\left(y_{i,exp}-y_{i,pred}\right)}^{2},$$

where $y_{i,exp}$ are the actual experimental values for the metric reported and $y_{i,pred}$ are the predicted values of the metric from the model.

## 4. Results and Discussion

### 4.1. Qualitative Analysis- Main Effects Plots

**Figure 4:**
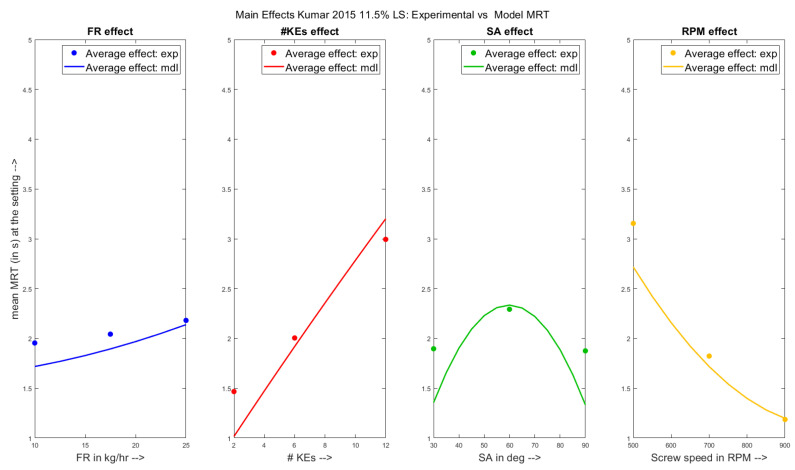
Experimental results vs. Model predictions: Main effects of varied parameters on MRT for the Kumar et al. 2015 [21] dataset.

The individual main effects of the varied parameters, powder feed rate (**FR**), number of kneading elements (**# KEs**) in the configuration, stagger angle between the kneading elements (**SA**) and the rotational speed of the shafts of the extruder (**RPM**) have been shown on the experimental MRT and on the model predicted MRT in Figure 4 for the Kumar et al. 2015 dataset. From the figures, it can be seen that the MRT of the TSG showed an increasing trend with respect to the increasing powder feed rate range from 10 kg/h to 25 kg/h and number of kneading elements from 2 to 12. On the other hand, the MRT decreased with respect to increasing screw speed from 500RPM to 900RPM. These observations are in line with the equations formulated in Section 3.2. However, it can be seen that the average MRT increased and then subsequently decreased as the stagger angle was increased from first 30${}^{\circ }$ to 60${}^{\circ }$ and then 90${}^{\circ }$. Additionally, it was reported by Kumar et al. 2015 [21], that several experimental data points were missing for run cases of 90${}^{\circ }$ SA due to the jamming of the equipment. This lack of comparable data points is the reason for a low average MRT value at high stagger angle.

Upon closer inspection of the main effects plots for the **FR** (indicated by the first sub-figure in blue), it can be seen that the model (solid curve) predicted a much steeper increase and wider range of MRT values compared to the experimental runs (individual points). The average model predictions were closer for powder feed rate values of 25 kg/h at higher MRT values. A similar observation is made from the second sub-figure (red curve, main-effects of **# KEs**) whereby, the average model prediction was lower compared to the experimental values at two kneading elements configurations yielding low MRT values. On the other hand, from the fourth sub-figure (yellow curve, main effects of **RPM**), the average MRTs predicted were lower at lower screw speeds corresponding to larger MRT settings. Lastly, it can be seen from the third sub-figure (green curve, main effects of **SA**) that MRTs were under-predicted at settings corresponding to lower experimental MRT (30${}^{\circ }$ & 90${}^{\circ }$**SA** values), and were over-predicting for the intermediate setting of 60${}^{\circ }$.

**Figure 5:**
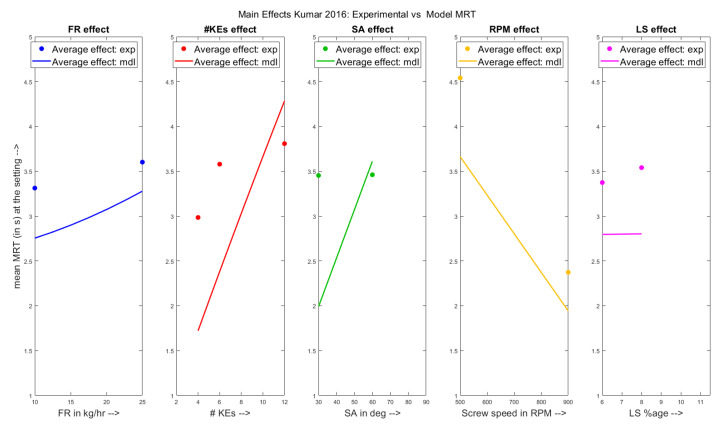
Experimental results vs. Model predictions: Main effects of varied parameters on MRT for the Kumar et al. 2016 [26] dataset.

The main effects of the varied parameters have been shown on the experimental MRT and on the model predicted MRT in Figure 5 for the Kumar et al. 2016 dataset. In addition to the previously shown effects (**FR**, **# KEs**, **SA** and **RPM**), there is an addiotional factor **LS** along which the effects have been in plotted too as indicated by the pink line and points. From the figures, it is seen that the MRT of the TSG showed an increasing trend with respect to the increasing powder feed rate range from 10 kg/h to 25 kg/h. On the other hand, the MRT decreased with respect to increasing screw speed from 500RPM to 900RPM. Similar to the results for the Kumar et al. 2015 dataset, these observations are in line with the equations developed. Comparing the main effects of the kneading elements, it can be seen that the model predicted average MRT values were lower than the experimental average values for 4 and 6 kneading elements in the screw configuration. The plausible reason for this observation may be that the granules formed had occupied more space in the kneading zone than what was calculated theoretically, thereby increasing the holdup and consequently the MRT. On the other hand, the mean experimental MRT showed no observable change upon increasing the stagger angle from 30${}^{\circ }$ to 60${}^{\circ }$. Similar to the effects of low number of kneading elements, the granules formed at 30${}^{\circ }$ stagger angle might have plausibly occupied more space than estimated leading to higher experimental MRT values. Despite these unexpected observations, the model equations were not changed to fit the predictions/main effects trends better as doing so would have removed the generalizable nature of the model which has been the goal of this study all along.

**Figure 6:**
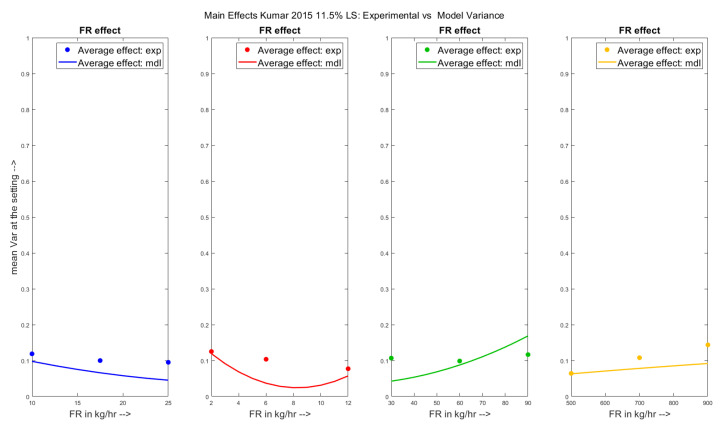
Experimental results vs. Model predictions: Main effects of varied parameters on variance for the Kumar et al. 2015 [21] dataset.

The main effects of the parameters have been shown on the experimental and the model predicted variance in Figure 6 for the Kumar et al. 2015 dataset. From the figures, it can be seen that the model predicted trends match the experimental trends for all the parameters except the stagger angle. It can be seen from the experimental trends for the Kumar et al. 2015 dataset only that increasing stagger angle did not effect the mean variance. However, the effect of stagger angle was still incorporated to predict the variance effects for the Kumar et al. 2016 dataset.

**Figure 7:**
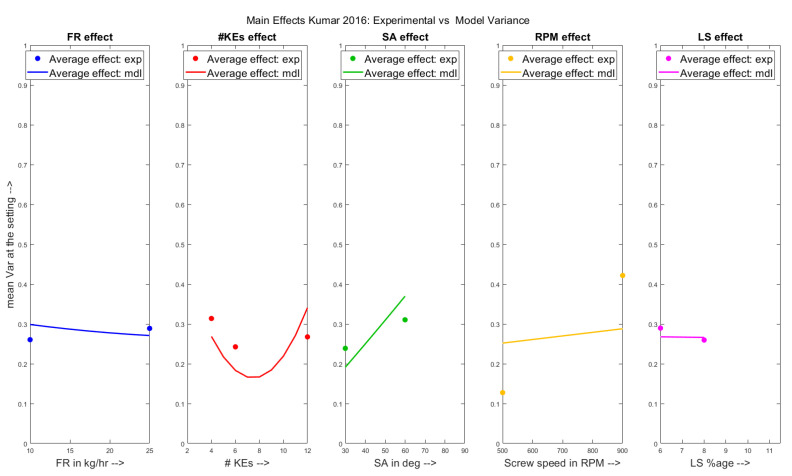
Experimental results vs. Model predictions: Main effects of varied parameters on variance for the Kumar et al. 2016 [26] dataset.

The main effects of the parameters have been shown on the experimental variance the model predicted MRT in Figure 7 respectively for the Kumar et al. 2016 dataset. From the figures, it can be seen that the model predicted a decreasing variance with increasing feed rate as was previously for the Kumar et al. 2015 dataset but the experimental trends showed a contradictory increasing trend. Another observation is that the experimental average variance increased on increasing the number of kneading elements in the screw configuration from 6 to 12.

### 4.2. Quantitative Analysis- Parity Plots

**Figure 8:**
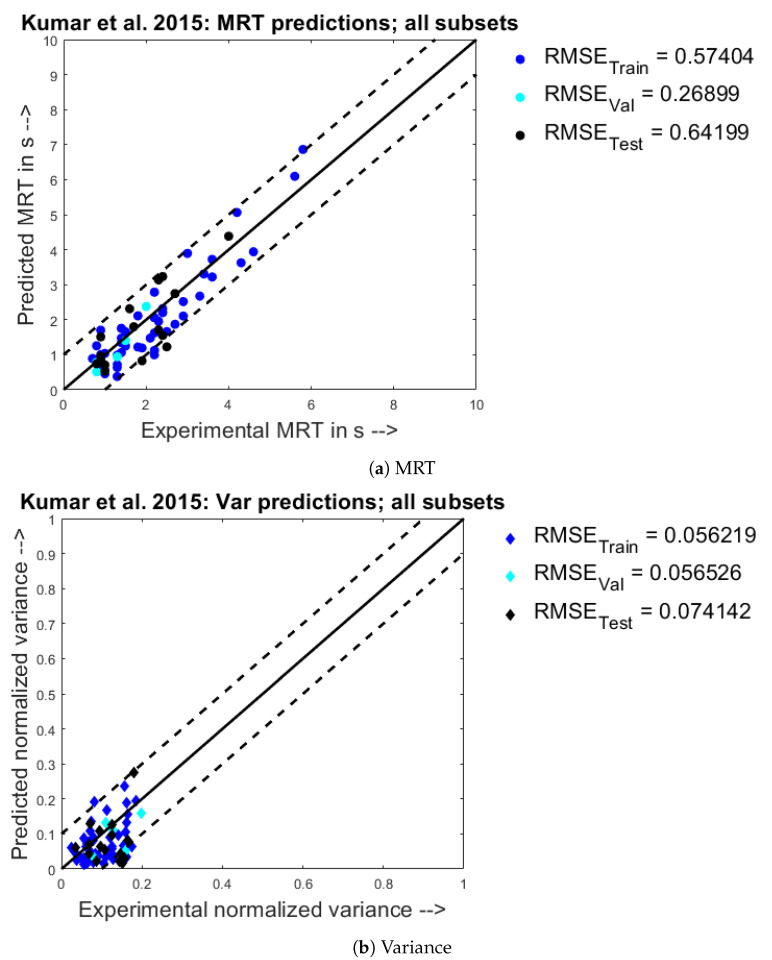
Experimental observations (X) vs. predicted model responses (Y) when trained on the Kumar et al. 2015 [21] dataset.

The parity plot for the experimental values of the MRT (X) vs. the predicted MRT values (Y) for the Kumar et al. 2015 dataset has been given below in Figure 8a. From the scatter points chart, it is seen that the most of the points were within the chosen confidence intervals of +/− 1 s. Moreover, it can be observed that most of the points are spread evenly on either side of the Y=X line. Similarly, from the parity plot for normalized variance for the Kumar et al. 2015 dataset in Figure 8b, it is seen that the most of the points were within the chosen confidence intervals of +/− 0.1, and most of the points are spread evenly on either side of the Y=X line. The confidence intervals were chosen in accordance with the variation of the points for the experimental dataset.

**Figure 9:**
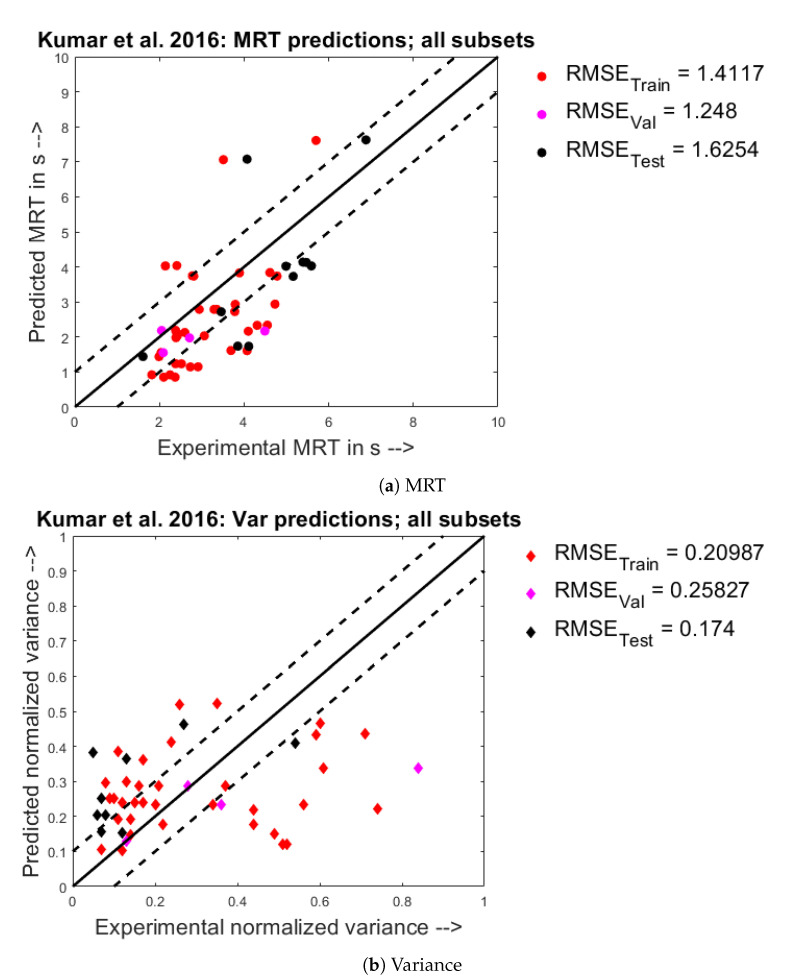
Experimental observations (X) vs. predicted model responses (Y) when trained on the Kumar et al. 2016 [26] dataset.

From Figure 9a, it is seen that the model under-predicts most of the experimental points under-predicted 1 second lower than their experimental value and lie on either side of the Y=X−1 line. Apart from this, it is also observed that a few points have been extremely over-predicted by as much as 75%. From Figure 9b, the model predictions for the normalized variance can be seen where, most of the points are scattered far away either side of the Y=X line.

**Figure 10:**
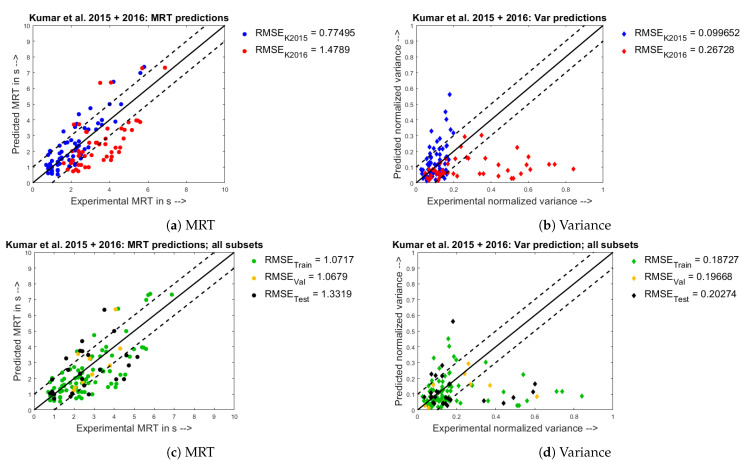
Experimental observations (X) vs. predicted model responses (Y) when trained on the combined Kumar et al. 2015 + 2016 [21,26] set.

The parity plots between experimental values and model predictions obtained by combining the two datasets, Kumar et al. 2015 and Kumar et al. 2016 are seen below in Figure 10. It is to be noted that the number of data-points from each individual dataset were in the same proportion in the total combined dataset and the partitioned datasets (Training, Validation & Test). Comparing the corresponding results for MRT and normalized variance, it is seen that the predictions built using a combined dataset are worse when compared to the predictions built using a single experimental dataset. It is seen that for the Kumar et al. 2015 dataset, the RMSE prediction using a jumbled training set is 0.77 s, and the RMSE for the Kumar et al. 2016 dataset is 1.48 s. Similarly, for the normalized variance results, the RMSE for the Kumar et al. 2015 dataset trained using a combined training dataset is 0.1, and the RMSE for the Kumar et al. 2016 dataset is 0.27. At this juncture, it must be noted that the LS% ages were 11.5% for the Kumar et al. 2015 dataset and the **LS**% ages were varied from 6 to 8% for the Kumar et al. 2016 dataset. One plausible reason for the poor prediction using the combined Training set may be that the main effects of **FR** have been contrary for the Kumar et al. 2015 and Kumar et al. 2016 experimental datasets respectively as seen in Section 4.1. Additionally, from the parity plots in Figure 10c,d, it is seen that the properly predicted, under- and over-predicted points were distributed in equal proportion across the training, validation and test datasets. This gives further indication to the veracity of the modelling approach.

**Figure 11:**
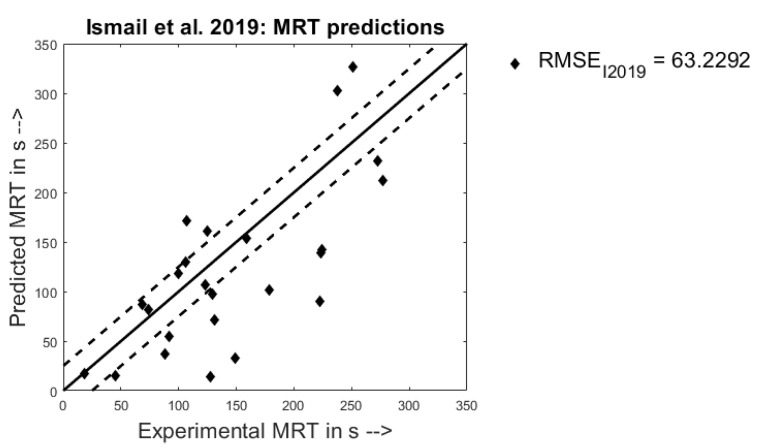
Experimental MRT observations (X) vs. predicted model MRT responses (Y) when trained on the Ismail et al. 2019 [14] dataset.

From Figure 11, it is seen that the model predicts the experimental values of the MRT for the Ismail et al. 2019 dataset in the right scale for quite a lot of the points, with many points +/− 20 s from their experimental value. However, there are quite a few points in the experimental values range of 125–225 s that have been under-predicted, thereby, leading to a large RMSE value of 63.23 s. It is to be noted here that the confidence interval for this dataset was higher compared to the previous datasets in order to account for the higher MRT values reported by Ismail et al. [14].

### 4.3. Quantitative Analysis- Comparing Model Parameters

The values of the fitting constants that were obtained by training the model on different datasets have been shown below in Table 2. Since the values $b_{2}$, $b_{3}$, $b_{4}$ and $b_{6}$ are exponential terms in Equations (9) and (11), it can be inferred that they indicate the extent of diminishing or magnifying effects of the corresponding physical terms incorporated in the factor. The effect of each constant would be to diminish if the value is less than 1, and magnify the factor if the value is greater than 1. On the other hand, the constants $b_{1}$ and $b_{5}$ are scaling parameters that aid in predicting the MRT and Peclet number/normalized variance in the right scale for each dataset. This is seen in the range of the MRT and normalized variance values respectively seen previously in Figure 8, Figure 9, Figure 10 and Figure 11. A similar behavior is seen in the exponent $b_{4}$ of the $Dispvolrate_{conv,1lead}$ as seen in the Equation (7).

**Figure 12:**
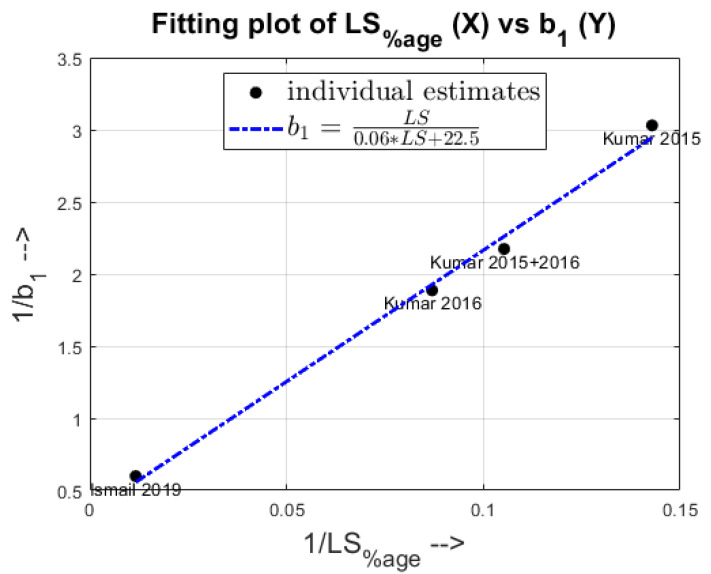
Fitting trend of $b_{1}$ for different datasets as the LS percentage is varied.

From Figure 12, it can be seen that the scaling constant for MRT fits with respect to varying liquid-to-solid percentages in a Langmuir-like trend. The reciprocal of the fitted parameter values, $b_{1}$ for each dataset was plotted against the respective reciprocal of the number-averaged LS percentage (Kumar et al. 2015- 7, Kumar et al. 2015 + 2016- 9.5, Kumar et al. 2016- 11.5 and Ismail et al. 2019- 85.83). From this trend we infer that as the liquid content in a TSG operation increases, the granulation rates would increase, thereby leading to greater quantities of larger granules in the system which would lead the particles to have a slower net average velocity giving a higher macroscopic MRT value. However, increasing the LS percentage beyond a limit would yield diminishing returns as the greater liquid quantities might just dissolve the excess powder particles and exit the system.

On the other hand, an additional effect is seen in the estimated values of the constants $b_{2}$, $b_{3}$ and $b_{6}$. The reasons for this observation would have to be further investigated by testing the model with more available datasets and checking the differences in the experimental conditions among them all.

## 5. Conclusions

From the main effects plots in Section 4.1, we could see that the same model relations yielded mostly similar trends across the datasets. This indicates the physical soundness and fundamental nature of the relations developed for predicting the measures of central moment, mean residence time (MRT) and Peclet number (Pe)/normalized variance. From the parity plots in Section 4.2, it is seen that the model performance varies with the distribution of the experimental data-points’ range. While the model predicted the experimental MRT and variance fairly well for the Kumar et al. 2015 dataset, however for several cases in the Kumar et al. 2016 and Ismail et al. 2016 dataset, there is either over- or under-prediction. A plausible explanation is that the prediction capability depends on the bounds of the changing process and equipment parameters for different experimental dataset combinations. Lastly, comparing the tuning parameter values for different datasets in Section 4.3, it is seen that they exhibit trends with respect to the inherent process complexities. One empirically inferred trend was found for the scaling constant $b_{1}$ for the MRT, and it was seen that the number-averaged liquid-to-solid percentage had a Langmuir effect on it. The presented approach and prediction models aim to serve as a starting point for future researchers to improve upon to obtain more mechanistic relations for the residence time central moment metrics, namely MRT and variance. Experimentally, having more points in a chosen design of experiments would also help in having more robust predictions by providing larger datasets to train and test the models upon.

Therefore, in summary, this research paper presents (i) a historical data-driven approach to estimating the RTD central moment metrics of a TSG system; (ii) a semi-mechanistic development of the predictive model relations (iii) validation of the said relations on different datasets; (iv) and lastly physical explanation for the values of the tuning parameters and empirical correlation for one of them with the liquid-to-solid percentage, one of the varying process parameters across datasets. 

## Figures and Tables

**Table 1:** Summary of the twin screw wet granulation residence time distribution (RTD) available and collected from the literature.

Data Source	Equipment Name	Process Material	Varied Parameters	Number of Points
Kumar et al. 2015 [21]	ConsiGma-25	α-Lactose MH	FR, RPM, NK & SA	66
Kumar et al. 2016 [26]	ConsiGma-25	α-Lactose MH	FR, RPM, LS, NK & SA	51
Ismail et al. 2019 [14]	Three-Tec	Avicel PH-101	FR, RPM, LS & NK	24
				**Total:** 141

**Table 2:** Model parameter values obtained for fitting on different datasets.

Parameter	Kumar et al. 2015	Kumar et al. 2015 + 2016	Kumar et al. 2016	Ismail et al. 2019
$b_{1}$	0.33	0.46	0.53	1.67
$b_{2}$	0.99	1.24	0.33	0.96
$b_{3}$	0.65	1.09	0.24	1.32
$b_{4}$	1.33	1.15	1.08	0.63
$b_{5}$	5.35	2.29	1.16	-
$b_{6}$	1.99	2.24	1.33	-

## Data Availability

The data presented in this study are available in: (1) Kumar et al. 2015 [21] Appendix A. supplementary material: 1-s2.0-S0928098715000548-mmc1.pdf. (2) Kumar et al. 2016 [26] Table S1. supplementary data: 1-s2.0-S0928098715300956-mmc1.pdf. (3) Ismail et al. 2019 [14] Appendix A. supplementary material: 1-s2.0-S0032591018309641-mmc1.xlsx and 1-s2.0-S0032591018309641-mmc2.xlsx.
